# Near-peer role modeling: Can fourth-year medical students, recognized for their humanism, enhance reflection among second-year students in a physical diagnosis course?

**DOI:** 10.3402/meo.v21.31940

**Published:** 2016-09-02

**Authors:** Mimi McEvoy, Staci Pollack, Lawrence Dyche, William Burton

**Affiliations:** 1 Department of Clinical Family and Social Medicine, Albert Einstein College of Medicine, Bronx, NY, USA; 2 Department of Pediatrics, Albert Einstein College of Medicine, Bronx, NY, USA; 3 Department of Clinical Obstetrics & Gynecology and Women's Health, Albert Einstein College of Medicine, Bronx, NY, USA

**Keywords:** reflection, peer feedback, near peer role modeling, humanism

## Abstract

**Introduction:**

Humanism is cultivated through reflection and self-awareness. We aimed to employ fourth-year medical students, recognized for their humanism, to facilitate reflective sessions for second-year medical students with the intention of positively influencing reflective process toward humanistic development.

**Methods/Analysis:**

A total of 186 students were randomly assigned to one of three comparison arms: eight groups of eight students (64 students) were facilitated by a fourth-year student who was a Gold Humanism Honor Society member (GHHS); eight groups (64 students) by a volunteer non-GHHS student; and seven groups (58 students) were non-facilitated. Before sessions, second-year students set learning goals concerning interactions with patients; fourth-year students received training materials on facilitation. Groups met twice during their 10 clinical site visits. At the last session, students completed a reflective assignment on their goal progress. Comparative mixed method analyses were conducted among the three comparison arms on reflection (reflective score on in-session assignment) and session satisfaction (survey) in addition to a thematic analysis of responses on the in-session assignment.

**Results:**

We found significant differences among all three comparison arms on students’ reflective scores (*p*=0.0003) and satisfaction (*p*=0.0001). *T*-tests comparing GHHS- and non-GHHS-facilitated groups showed significantly higher mean reflective scores for GHHS-facilitated groups (*p*=0.033); there were no differences on session satisfaction. Thematic analysis of students’ reflections showed attempts at self-examination, but lacked depth in addressing emotions. There was a common focus on achieving comfort and confidence in clinical skills performance.

**Discussion/Conclusions:**

Near peers, recognized for their humanism, demonstrated significant influence in deepening medical students’ reflections surrounding patient interactions or humanistic development. Overall, students preferred facilitated to non-facilitated peer feedback forums. This model holds promise for enhancing self-reflection in medical education, but needs further exploration to determine behavioral effects.

Humanism, the simple regard of one person for another, is a common casualty in the mechanistic, rushed, and data-driven world of modern medicine. Preserving humanism in such an impersonal context requires vigilant self-reflection and positive role modeling (1–3). Medical humanism, once in danger of becoming a stepchild of technology, is now regaining a secure place in the training of physicians by virtue of its relationship to patient-centered medicine (4), effective communication (5), and medical error management (6). Humanism is regarded as a pillar of professionalism (7) and, as such, is supported by the ACGME as a core competency in medical education (8).

Since self-awareness is essential to humanism, many approaches have been developed to foster self-reflection in medical students (9), but current literature on the poor reliability of independent self-assessment (10, 11) is indicating that the skill of ‘feedback seeking’ is an essential component of the capacity to reflect (12). Feedback from peers and near peers (students from later years) is particularly important for acquiring accurate self-assessment and developing professionalism because peers presumably have a view of one another that is often more accurate than faculty observation. The near-peer participants in this study who facilitated the feedback forums of second-year students in the physical diagnosis course were fourth-year medical students, half of whom were members of our chapter of the Gold Humanism Honor Society (GHHS). The students in this society are selected by their peers for emulating humanistic values including altruism, compassion, and honesty and thus, held as exemplary role models.

## Definitions of reflection and self-assessment

The concepts of reflection and self-assessment are used in tandem in this article. Each are essential steps in the process of professional identity formation (13) of which Stern includes humanism as an element of the larger construct of professionalism (14). Reflection helps learners form assumptions about themselves for which they can seek validation through external feedback. Self-assessment is a process in which the student evaluates the understanding achieved through reflection and can set personal and professional goals for learning and improvement.

## Description of the clinical examination course

*The Clinical Examination* course, traditionally known as ‘physical diagnosis’, is one of three courses within our larger *Introduction to Clinical Medicine (ICM) Program*, which spans the first and second year of medical school. The course is comprised of 186 medical students who work in pairs to elicit a patient's history and perform a physical examination over 10 sessions between January and May each year. Pairs of students split the tasks of history-taking and physical examination each week (one student elicits the history; the other performs the physical examination) and switch tasks the following week. This model provides ample opportunity for students to observe each other's skills and behaviors in these patient settings. In addition, student pairs are precepted at the bedside by the same attending physician or a physician pair for all 10 clinical site visits.

## Methods

### Project design

As part of the physical diagnosis course in the 2014/2015 academic year, we initiated two 90-minute-facilitated reflective sessions that occurred on the medical school campus – after the fifth and ninth clinical site visits. In preparation for these sessions, we required students to formulate personal learning goals regarding their interactions with patients at the clinical sites after the second clinical site visit. In addition, we required students to share their goals with their practice partners, observe each other's skills and interactions with patients, and give informal, ongoing feedback to each other during or immediately after the site visits.

For the two sessions, student pairs (*n=*186) were randomly assigned to one of three comparison arms: eight groups of four practice pairs (*n=*64) were facilitated by a fourth-year student who was a GHHS student; eight groups of four practice pairs (*n=*64) by a non-GHHS student; seven groups of four practice pairs, one group having five pairs (*n=*58) were non-facilitated; this later comparison arm was formed to control for the effect of facilitation alone.

The fourth-year students were volunteers and recruited on a first-come-first serve basis (GHHS and non-GHHS students); they received no incentive other than being given a lunch voucher for the cafeteria after each of the two sessions. These sessions were conducted at the medical school and did not involve their clinical site preceptors. All student facilitators (GHHS students and non-GHHS students) attended together a 60-minute training session with one of the authors (LD), a social worker experienced in small group teaching and learning. In these facilitator trainings, we emphasized process rather than content surmising that the personal values of the facilitators would be the influencing factor. Since the goal of the two sessions was to generate an open discussion with feedback seeking from peers, the role of the facilitator was to help student pairs process their learning goals set earlier in the course about their interactions with patients and generate feedback sharing in the groups. We assumed that these near-peer facilitators would interject comments and responses at various times during these sessions and that their innate humanistic characteristics and values about patients and patient interactions would be revealed and have some effect on the students. This humanistic influence is the ingredient which we hypothesized would be apparent in the end of course reflective assignment.

In this educational research study design, we used a comparative, mixed method to measure the influence of GHHS students on their younger peers regarding the process of professional identity formation. We hypothesized that the GHHS students would be more influential than the non-GHHS students with regard to students’ reflective capacity by virtue of their noted intrinsic humanistic characteristics.

This study was deemed exempt by our Institutional Review Board.

### Goal-setting reflective exercise and the reflective process

All reflective sessions were 90 minutes long and met at the medical school twice during the 10-session course. After the second clinical site visit, all students completed a goal-setting assignment, which required them to set personal and professional learning goals for their work with patients at the clinical sites. This goal-setting exercise served as a prompt for students for the first reflective session to become cognizant of their thoughts, behavior, and attitudes regarding humanism as well as to sensitize them to observing and commenting on the humanistic behavior of their peers.

**Table 1:** In-session reflective assignment

What progress do you feel you have made with the personal goal you set at the beginning of the year? How do you measure this progress? Is there more that you might do to advance the goal? How did you deal with the personal challenges you anticipated in pursuing this goal? Were there pivotal events or people that helped you? What do you see as an appropriate goal for next year and why?

The first reflective session occurred after the fifth clinical site visit. In the session, we asked students convened in the small groups described earlier to review and reflect on their goals, what progress they had made, why and what implications this held for setting goals later on. A key component of the reflective process in these sessions was to encourage students to solicit feedback regarding the progress they had made on their goals from their peers in these groups. After the ninth clinical site visit, we sent students an email in preparation for the second reflective session. We asked them to revisit the goals that they set for themselves earlier in the course (reflective goal-setting assignment) and had discussed in the first reflective session. Further, we asked students to consider the progress they made during the course toward these goals and to exchange their goals with their clinical site partners. We expected that students would come prepared to comment not only on the progress of the goals that they had set for themselves, but to give feedback to their clinical practice partner as well. Students were instructed to bring either a hard copy or electronic access of their reflective goal-setting assignment to the session for reference. At the end of this second session, students completed an in-session written reflective assignment on the goal progress they had made during the course (see Table 1) and complete a satisfaction survey item, ‘Rate on a scale of 1–4 your overall impression of the usefulness of these two sessions’. Rating scale was 1=‘not useful’; 2=‘somewhat useful’; 3=‘useful’ and 4=‘very useful’.

### Analysis

#### Developing the in-session written reflection scoring rubric

In developing a rubric to analyze the in-session written reflections, we first explored the literature on scoring levels of reflective writing (15). Since most reflection assessment rubrics discussed in the literature concern writings about sentinel events or personal experiences, our focus on goal setting and self-assessment required us to adapt existing instruments to fit the needs of our study.

In our survey, we noted that most rubrics consider whether the authors identified any personal challenge in their narratives; the degree to which they demonstrated self-analysis or considered external feedback; and, apropos the matter of humanism, whether the authors explicitly considered the emotions engendered either within themselves or their patients.

In reviewing the in-session reflections, we noticed that the majority of students specified a plan to ‘practice’ taking history and doing physical exams frequently enough to gain a level of ‘comfort’ and ‘confidence’. Because these expressions were nearly ubiquitous as students recalled the goals they set at the beginning of the year, we decided to use them as a baseline for reflection and award them *one point*. We awarded them an additional point if the student elaborated: if the student specified additional challenges beyond those in the baseline, we awarded them *two points* for reflection, for example, ‘Ï want to become as efficient as possible’. If the student undertook a degree of self-analysis, ‘Ï know I tend to go off on tangents with a patient, and I must learn to curb this habit’, we awarded the student *three points* for reflection. Finally, if the student alluded to their own emotions or those of their patient, we awarded them *four points* for reflection, for example, ‘When I'm anxious, I can talk too much as I examine a patient; and I think it makes them uneasy as well’. The affective component was given the highest status because we saw human emotion as a critical aspect of the humanistic perspective that is often overlooked in medicine (see Table 2 for a description of each reflective level).

**Table 2:** Rubric for scoring the reflective exercise

Level of reflection		Description of reflective level	Discrimination challenges
Level 1	No reflection	No statement of challenge or problem beyond ‘needing time/practice to become comfortable/confident/proficient’. I'm doing fine and expect to continue to do so.	What is an adequate challenge or problem? Anything more than getting comfortable?
Level 2	Goal setting without reflection	Student at this level can specify a particular challenge, asking difficult questions or focusing the exam, but does not relate it to any personal issue or attempt at self-understanding.	What is an ‘adequate’ level of self-examination? Is it quantitative or qualitative?
Level 3	Reflection that falls short of accessing feelings	Survey shows more than one attempt at self-examination in order to understand the problem or seek a solution. However, this effort is at a cognitive level and does not extend to feelings.	What constitutes a feeling reference? How direct and literal must it be?
Level 4	Cognitive/affective reflection	At this level the survey contains references to the feelings of the author or of others. These may be stated, for example, afraid or embarrassed, or they may be implied, for example, a patient being able to open up helped me learn to ask sensitive questions more easily.	Does integrative simply mean refers to future at both cognitive and feeling levels?

Students evaluated the sessions after the second or last session by completing a satisfaction survey using a 4-point Likert-type scale regarding the sessions’ usefulness in attending to personal goal setting and development.

#### Scoring the written reflections

Two of the authors with experience in qualitative analysis randomly selected 20 reflection papers (11%), scored them independently, and used our differences to identify and improve agreement. Following this process, the same two authors scored a second random selection of 20 reflection papers (11%) and achieved greater consensus. We scored an additional 35 papers independently (19%), which yielded a correlation coefficient of 0.858. The remaining 110 reflection papers were equally divided between the two authors and scored. After these scores were recorded, the third author read a random sample of 25 papers to determine independent agreement with scoring. We applied ANOVA to compare the reflective scores and satisfaction with the sessions among the three groups – GHHS-facilitated, non-GHHS-facilitated, and non-facilitated groups.

## Results

These reflective sessions were an element of the required curriculum, and thus, 182 of 186 students participated (four students were excused from the actual session), yielding a 98% response rate. We found significant differences among all three comparison arms regarding their in-session reflective assignment scores as well as their satisfaction with the group process (see Table 3)**. In addition, we found that students in groups led by near peers who were noted for their humanism (GHHS members) had higher reflective scores compared to students in groups led by near peers who were not GHHS members (mean=2.56 vs. 2.21, respectively; *p=*0.033). Despite differences in reflective scores between the GHHS lead and non-GHHS lead groups, there was no difference in students’ level of satisfaction. Differences in satisfaction among all three arms were highly significant indicating that students preferred facilitated groups as opposed to having a non-designated facilitator (see Table 4)**.

**Table 3:** Comparison of groups on reflective score

Group	*N*	Reflective score	SD
A	45	2.56	0.81
B	57	2.21	0.80
C	49	1.82	0.97

Analysis completed using ANOVA; *p*=0.0003; mean scores on a 4-point scale.

**Table 4:** Comparison of groups on satisfaction with reflective sessions

Group	*N*	Satisfaction score	SD
A	58	2.24	0.92
B	56	2.23	0.85
C	48	1.52	0.68

Analysis completed using ANOVA; *p*=0.0001; mean scores on a 4-point scale.

Thematic analysis of the in-session reflections among students in all three comparison arms provided an opportunity for us to describe or characterize students at various stages of reflective ability. Level 1 reflectors only expressed preference for practice to achieve comfort and confidence in doing the physical diagnosis interview and examination. While the level 2 reflectors noted additional challenges, they still did not demonstrate any self-reflection. Seventy-five percent of these challenges did not involve the patient except as object in the concern. Students whose reflective ability reached level 3 demonstrated an effort at self-examination, but did not allude to their own or their patient's feelings. These reflections were split between those who sought understanding by looking within themselves and those who sought confirmation from those around them. Finally, level 4 reflectors alluded to feelings in their reflections, some of which concerned their own sense of awkwardness and vulnerability, others attending to patient comfort and understanding.

## Discussion

This study sought to determine if near-peer role modeling in reflective sessions led by GHHS students could influence reflection levels, measured by the authors’ rubric, among groups of second-year medical students discussing their goals for professional and humanistic development during their physical examination course. The findings demonstrated significantly higher levels of reflection by students in the GHHS student-facilitated groups when compared with control groups facilitated by non-GHHS near peers and in groups without a facilitator. Further, the absence of a facilitator resulted in a marked reduction in reflection levels.

While student satisfaction with the groups was significantly lower in non-facilitated groups, the satisfaction between students in the GHHS- and non-GHHS-facilitated cohorts was not significant. Thus, the GHHS students produced groups that were more deeply reflective, but not more satisfied with their group experience.

The finding pertaining to reflective ability was reassuring to the authors, because we feared the design necessitated by the course logistics produced such a low-dose intervention that differences in the two facilitated groups might be undetectable or insignificant. Since this was not the case, our speculation is that not only did the GHHS facilitators demonstrate a different ethic than those who had not earned the reward but were also working with their students using concepts and a vocabulary more conducive to the humanism reflection measured by the rubric.

The finding that student satisfaction with the group experience did not correspond to the depth of reflection students achieved suggests that reflection and satisfaction are separate and unrelated dimensions, or else that the work of reflection carries a burden that might suppress group member satisfaction.

In the qualitative domain, it is striking that the vast majority of students specified goals of ‘practice, confidence, and comfort’ especially at the lower levels of reflection scoring. If these are interpreted as student concerns about themselves to the exclusion of their patients, it would indicate a diminished level of humanism or possibly an indication of the stress involved in patient encounters at this early level of training. But, these possibilities need further qualitative exploration before any assumptions can be made.

The explanation for students’ strong preference for group facilitation might be self-evident – that students prefer leadership in the work of feedback and reflection. While we found a significant difference among groups regarding depth of reflection and session satisfaction, the overall mean scores for both measures were low (2.56/4.0–1.82/4.0 and 2.24/4.0–1.52/4.0, respectively). Explanations for the overall low reflective scores might have resulted from a limitation of the rubric in being able to discriminate adequately between reflective levels, a tendency to assign a lower reflective score if students focused solely on clinical skills development despite a deeper reason for their discomfort that wasn't captured in the rubric. Or, the anxiety in mastering the skills overshadowed their abilities to more deeply reflect on the intricacies and nuances of the doctor–patient interactions. This possibility is supported by data gleaned from a series of fourth-year students’ focus groups revealing that ‘stressful conditions’ inhibited their humanism (16), which in our case might have been the focus of basic clinical skills development.

Limits of the study include the use of an un-validated rubric that required several trials in order to achieve satisfactory inter-rater concordance. The area of data analysis that proved most difficult for us concerned discriminating a level 2 from a level 3 reflection: essentially determining what constituted adequate self-analysis. There was also a possibility of a level 2 alluding to emotions without self-analysis, although the case never arose.

Another limitation is that we did not collect any baseline data on individual students regarding their humanistic characteristics that could have affected their ability to benefit from our intervention and which would have provided more meaningful information. Additionally, aside from anecdotal comments, we did not collect any information from the near-peer facilitators (fourth-year students) that might have explained some of the factors influencing the differences in reflection among the comparison groups and session satisfaction. Finally, our study was conducted at one institution, and therefore, the results are not generalizable.

## Conclusions and future plans

We concluded that near peers, recognized for their humanism, demonstrated a significant influence in deepening medical students’ reflections surrounding patient interactions. In addition, students prefer facilitated to non-facilitated peer feedback forums. This model holds promise for enhancing self-reflection in medical education, but needs further exploration to determine behavioral effects. Future plans also include conducting focus groups of facilitators after these reflective sessions in order to better understand the factors contributing to enhancing reflection surrounding humanistic development or professional identity formation as well as overall satisfaction with the process.

## References

[1] Mann K, Gordon J, MacLeod A (2009). Reflection and reflective practice in health professions education: a systematic review. Adv Health Sci Educ Theory Pract.

[2] 
Gracey CF, Haidet P, Branch WT, Weissmann P, Kern DE, Mitchell G (2005). Precepting Humanism: strategies for fostering the human dimensions of care in ambulatory settings. Acad Med.

[3] Weissmann PF, Branch WT, Gracey CF, Haidet P, Frankel RM (2006). Role modeling humanistic behavior: learning bedside manner from the experts. Acad Med.

[4] Smith RC, Hoppe RB (1991). The patient's story: integrating the patient and physician-centered approaches to interviewing. Ann Intern Med.

[5] Haidet P, Paternini DA (2003). Building a history rather than taking one: a perspective on the information sharing during the medical interview. Arch Intern Med.

[6] Mamede S, Schmidt HG, Penaforte JC (2008). Effects of reflective practice on the accuracy of medical diagnosis. Med Educ.

[7] Arnold L, Stern DT, Stern DT (2006). What is medical professionalism. Measuring medical professionalism.

[8] ACGME Core Competencies Program requirements http://www.acgme.org/acgmeweb/Portals/0/PFAssets/ProgramRequirements/CPRs2013.pdf.

[9] Sanders J (2009). The use of reflection in medical education: AMEE Guide No. 44. Med Teach.

[10] Langendyk V (2006). Not knowing that they do not know: self-assessment accuracy of third-year medical students. Med Educ.

[11] Carr SE, Johnson PH (2013). Does self-reflection and insight correlate with academic performance in medical students?. BMC Med Educ.

[12] Milan F, Dyche L, Fletcher J (2011). ‘How am I doing?’: teaching medical students to elicit feedback during their clerkships. Med Teach.

[13] Cooke M, Irby DM, O'Brien C (2010). Educating physicians-A call for reform of medical school and residency.

[14] Stern DT (2006). Measuring medical professionalism.

[15] Wald HS, Borkan JM, Taylor JS, Anthony D, Reis SP (2012). Fostering and evaluating reflective capacity in medical education: developing the REFLECT rubric for assessing reflective writing. Acad Med.

[16] Moyer CA, Arnold L, Quaintance J, Braddock C, Spickard A, Wilson D (2010). What factors create a humanistic doctor? A nationwide survey of fourth-year medical students. Acad Med.

